# Esophageal Paget’s Disease Secondary to Hypopharyngeal Carcinoma: a Case Report

**DOI:** 10.1007/s12029-018-0087-2

**Published:** 2018-03-15

**Authors:** Holly White, Simi George, James Gossage, Fuju Chang

**Affiliations:** 1 Department of Histopathology, Guy’s and St Thomas’ Hospitals NHS Foundation Trust, London, UK; 2 Cellular Pathology Department, Royal Surrey County Hospital, Guildford, UK; 3 Department of Surgery, Guy’s & St Thomas’ Esophago-Gastric Centre, London, UK; 4 School of Cancer and Pharmaceutical Sciences, King’s College London, London, UK

## Introduction

Paget’s disease is defined as neoplastic cells of glandular differentiation infiltrating squamous epithelium [1]. In essence, it represents a population of malignant mucin-producing cells which sit in amongst benign squamous cells within skin or mucosa. These cells can arise secondary to a primary carcinoma, which may have so called ‘Pagetoid spread’ of malignant cells at the periphery of the lesion, or more rarely, Paget’s cells can arise de novo within the squamous epithelium, the pathogenesis of which is not yet understood. Having been first described in the breast by Sir James Paget in 1874, extramammary Paget’s disease is an entity most commonly seen in the vulva and anus but it is known to occur rarely in the esophagus, almost exclusively in the context of invasive esophageal adenocarcinoma. Paget’s cells within esophageal squamous epithelium are found in 4.9% of invasive adenocarcinoma cases and are most commonly associated with diffuse, poorly differentiated adenocarcinoma with dyscohesive malignant cells [2–5]. Few case reports are present in the literature to describe Paget’s cells within the esophagus but those that exist include cases involving mucous gland carcinoma, adenosquamous carcinoma and adenocarcinoma arising on a background of Barrett’s esophagus [2–5].

We present a case in which widespread esophageal Paget’s disease arises secondary to poorly differentiated carcinoma of the hypopharynx. A literature search did not yield any similar cases.

## Case Report

**Fig. 1:**
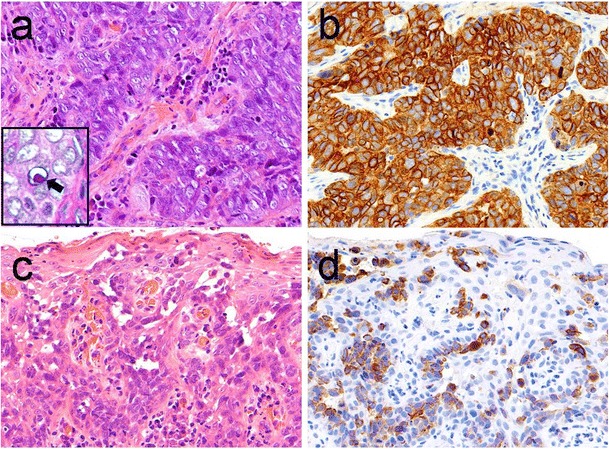
Biopsy from right piriform fossa of the hypopharynx shows grade 3, poorly differentiated carcinoma (**a**) which is positive for CK7 (**b**). There is pagetoid spread in the overlying squamous epithelium (**c**). The Paget’s cells are also positive for CK7 (**d**). Insert: alcian blue diastase-PAS highlights the cytoplasmic mucin within the carcinoma cells

The patient was a 63-year-old gentleman, a retired car mechanic, with a known history of T1N0M0 grade 3 carcinoma of the right piriform fossa of the hypopharynx diagnosed in 2015 for which he underwent surgery and radiotherapy. The histology from the primary hypopharyngeal lesion showed an invasive, poorly differentiated carcinoma with adjacent Paget’s disease (Fig. 1). The invasive carcinoma and the adjacent Paget’s cells were found to show positive immunohistochemical staining for CK7 and EMA, patchy positivity for BerEP4 and p16 and negative staining for p63, CK20, CEA, Bcl-2 and S100. This immunoprofile is consistent with a poorly differentiated adenocarcinoma. Alcian blue diastase-PAS highlighted the cytoplasmic mucin within the carcinoma cells (Fig. 1). Whilst the invasive carcinoma had been completely excised, the pagetoid lesion was present at the resection margin.

Upper gastrointestinal endoscopy and esophageal biopsies were carried out following the excision of the primary lesion and the Paget’s disease was found to extend into the esophagus, prompting regular follow-up endoscopies and biopsies.

**Fig. 2:**
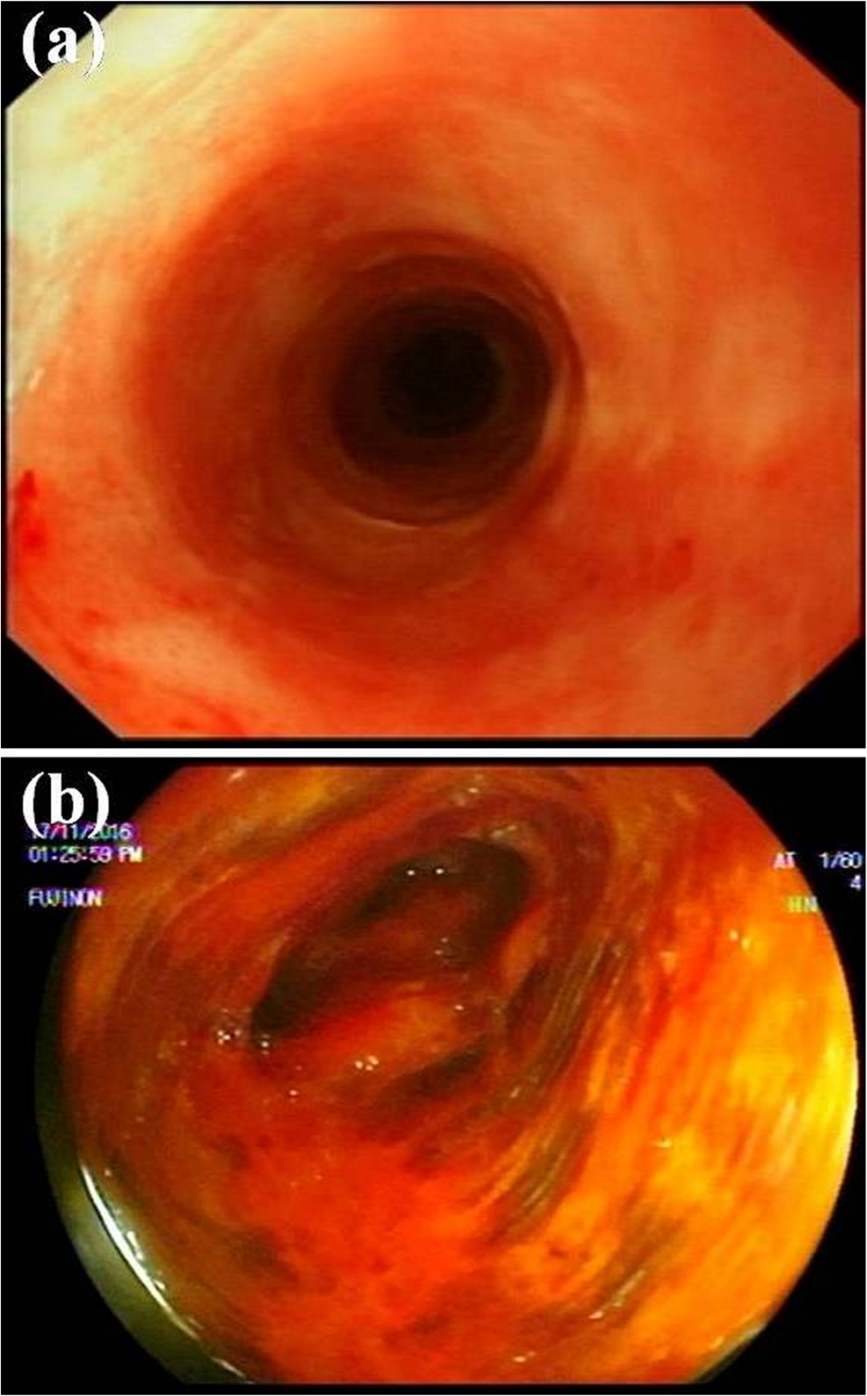
Preoperative white light endoscopy shows erythematous esophageal mucosa but no nodules or mass lesions (**a**). On Lugol’s iodine chromoendoscopy, the whole esophagus was unstained, with only a few patches of normal staining (**b**)

Following the last endoscopy, which yielded a diffusely abnormal Lugol’s iodine test (Fig. 2), the multidisciplinary team made the decision to undertake an esophagectomy amidst concerns regarding possible progression to invasive carcinoma. Biopsies taken during this last endoscopy showed similar features to the Paget’s disease found adjacent to the primary hypopharyngeal lesion, namely, infiltration of the squamous epithelium of the esophagus by carcinoma cells with enlarged nuclei and prominent nucleoli along with positive immunohistochemical staining for CK7 and EMA and negative staining for p63, CK20 or S100.

**Fig. 3:**
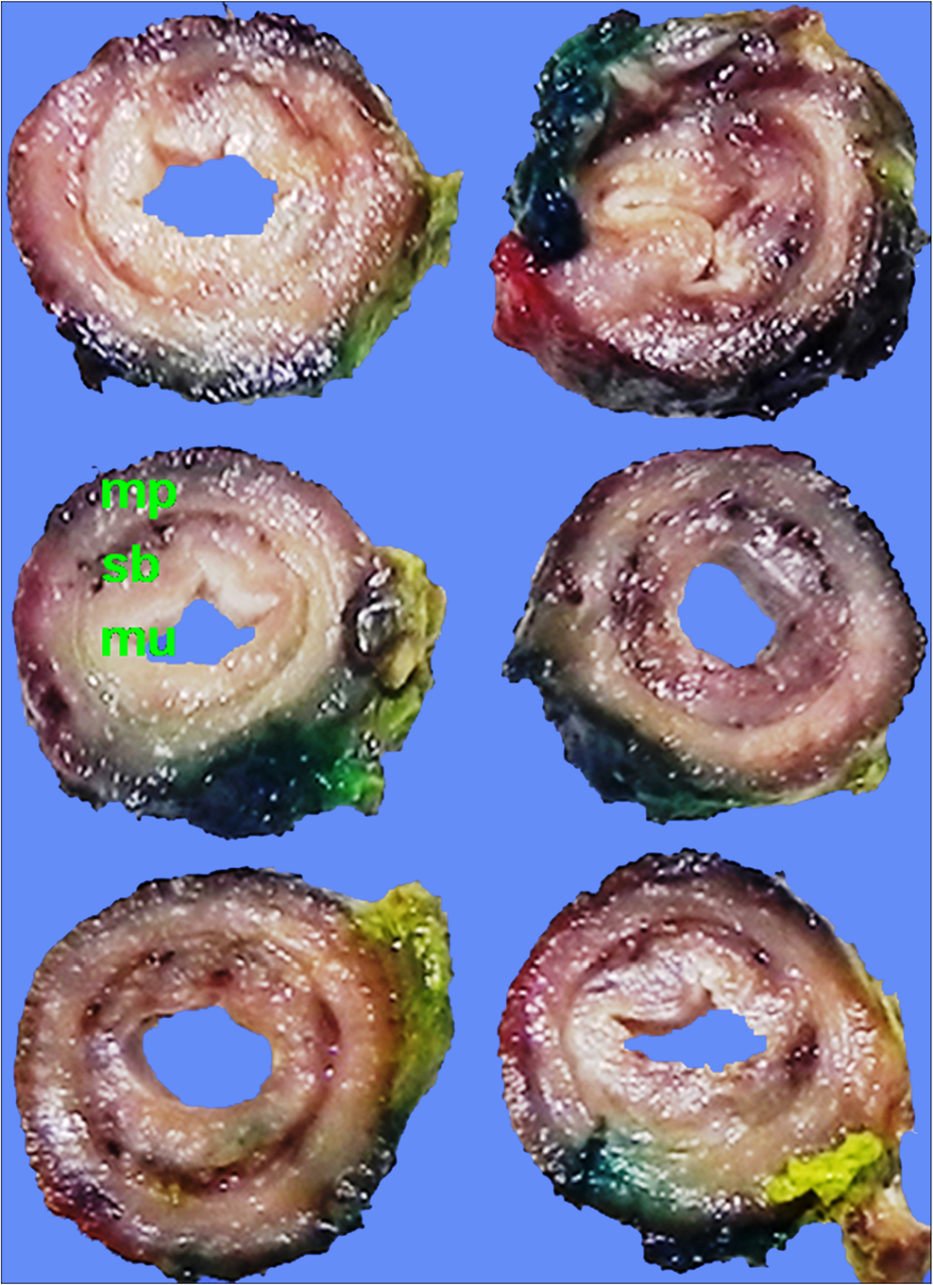
Esophagectomy specimen sliced transversely. The specimen shows a grossly normal architecture with unremarkable mucosa (*mu*), submucosa (*sm*) and muscularis propria (*mp*). No mass lesions are seen macroscopically

**Fig. 4:**
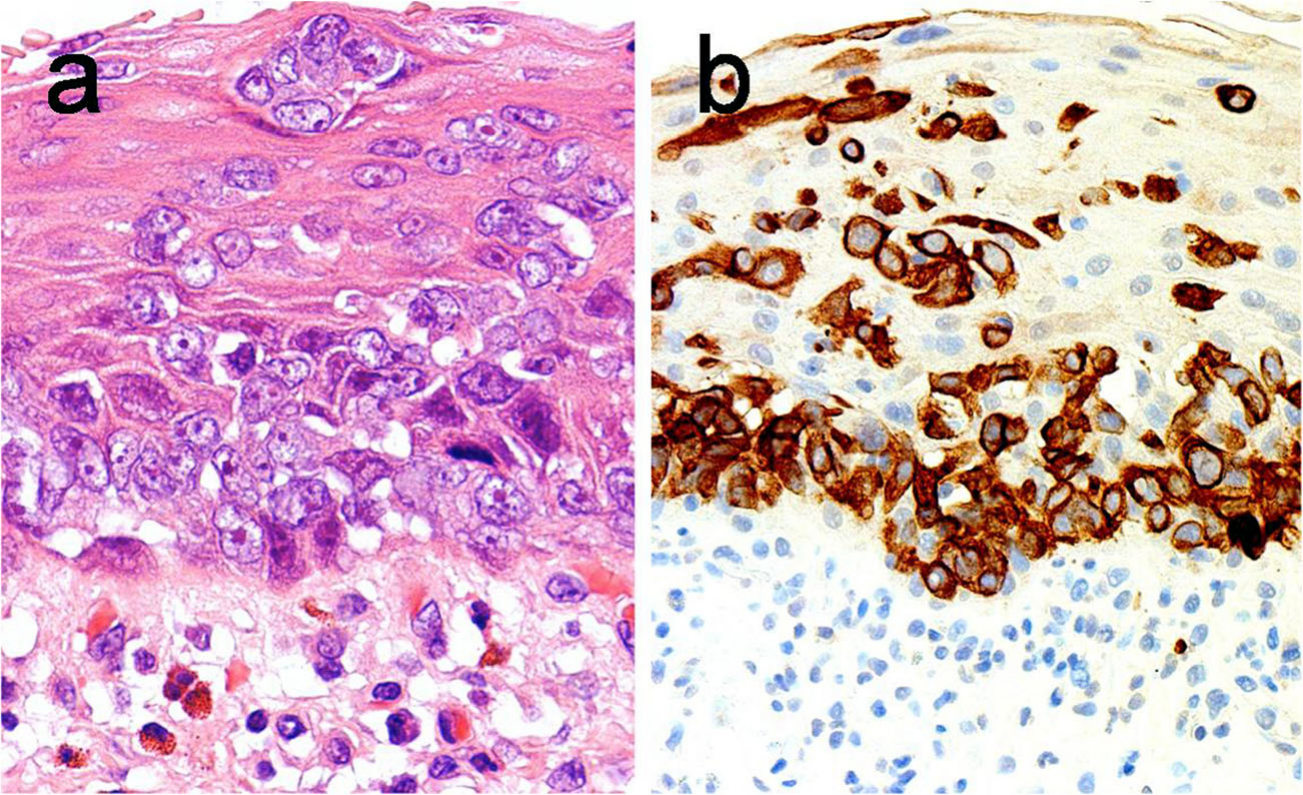
Paget’s disease of the esophagus. On microscopic examination, the surface squamous epithelium shows extensive infiltration by large atypical cells (**a**). These cells are positive for CK7 (**b**)

**Fig. 5:**
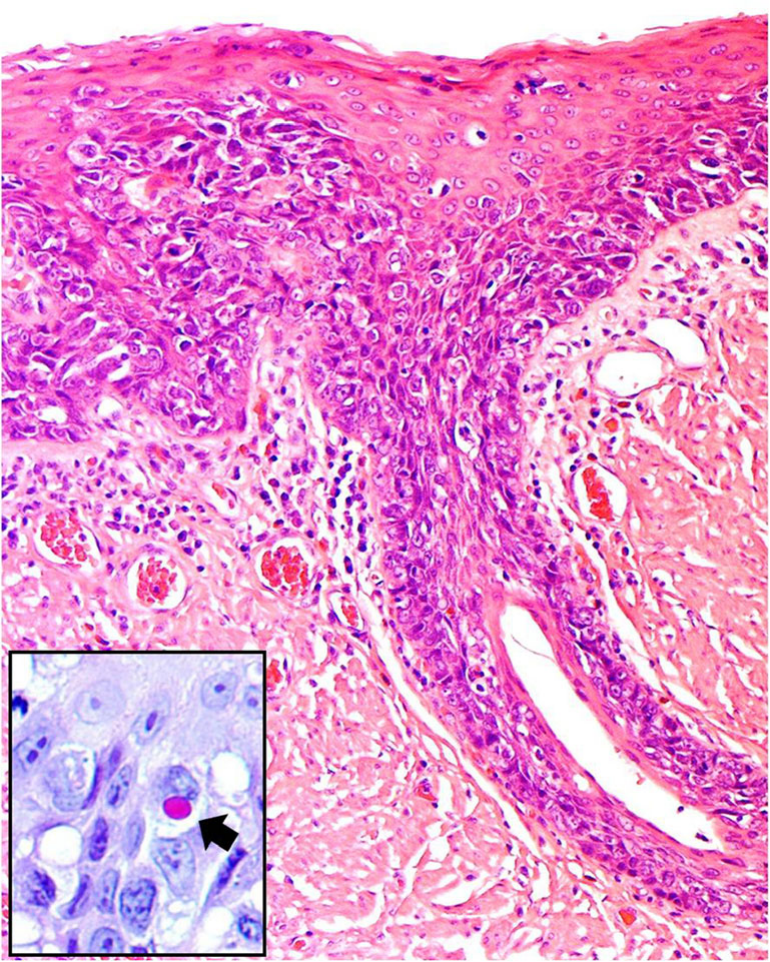
Paget’s disease of the esophagus. Intraepithelial infiltration by large atypical Paget’s cells is seen in surface squamous epithelium and mucus gland ducts. Insert: alcian blue diastase-PAS highlights the cytoplasmic mucin within the Paget’s cells

A total esophagectomy was performed and macroscopic examination found an esophagus measuring 140 mm × 25 mm × 15 mm with an attached portion of stomach measuring 92 mm along the lesser curve and 158 mm along the distal greater curve staple line. Serial slicing of the esophagus showed a grossly normal mucosal surface (Fig. 3). No mass lesions were identified. Microscopic examination of the extensively sampled esophagus revealed similar features to the previous biopsy in that there were widespread Paget’s cells infiltrating throughout the squamous epithelium (Fig. 4), with a lack of any invasion of the basement membrane. This was present throughout the entire length of the esophagus and involved the esophageal ducts (Fig. 5) as well as the proximal resection margin. The large, hyperchromatic, atypical cells in the esophagectomy specimen were found to be positive for CK7 and EMA (Fig. 3) whilst being negative for CK20, CK5, p63, S100, CDX2, TTF1 and PSA. Special staining with alcian blue diastase-PAS highlighted the cytoplasmic mucin within the Paget’s cells (Fig. 5). No invasive adenocarcinoma was identified throughout the esophagus.

We feel that this is a rare case of Paget’s disease extending from a primary lesion site within the hypopharynx into the distal part of the esophagus.

## Discussion

Paget’s disease is a recognized, although rare, entity in the esophagus, almost exclusively seen in the presence of co-existing esophageal adenocarcinoma. A study of esophageal adenocarcinoma cases carried out in 2007 by Abraham et al. found that Paget’s disease is present in 4.9% of such cases [1]. Amongst the eight patients in the study identified as having esophageal Paget’s disease, seven were male and one was female, with a mean age of 62.4 years, demographics which mirror those of esophageal adenocarcinoma. The study found that five of these eight cases had not previously been reported as showing Paget’s cells, which was only noted on subsequent review as part of the study. This might suggest that Paget’s cells can be overlooked during the initial assessment of esophageal adenocarcinoma specimens. The same study also found that the most useful special stain for highlighting the intracellular mucin associated with Paget’s disease was PAS-diastase [1]. The glandular differentiation of Paget’s cells can be revealed by positive immunohistochemical staining for glandular cytokeratins (CK7), EMA and CEA. Notably, EMA (also called MUC1) is universally expressed by nearly all cases of extramammary Paget’s disease and it is comparable with CK7 as a universal cytokeratin marker for Paget’s cells [6].

Our case of esophageal Paget’s disease arises in the absence of a primary esophageal lesion, but secondary to a poorly differentiated hypopharyngeal tumour, with the pagetoid spread of malignant cells extending the entire length of the esophagus. A literature search did not find any other similar cases, although there are two cases reported of primary Paget’s disease of the esophagus, in which the Paget’s cells were thought to have arisen de novo within the squamous epithelium [2, 3]. These cases, similarly to ours, arose in the absence of a primary esophageal lesion, although there was no mention of a primary pharyngeal tumor in these reports.

An important, although extremely rare, differential in cases of Paget’s disease of the esophagus is melanoma. The dyscohesive, large, atypical cells distributed throughout the squamous epithelium is typical of pagetoid spread by malignant melanoma which accounts for 0.1–0.2% of primary esophageal malignancies [7]. For this reason, melanocytic markers are an important component of the assessment of these cases.

In light of this case, it may be prudent to perform regular upper gastrointestinal endoscopy and esophageal biopsies in patients who are found to have Paget’s disease of the epithelium adjacent to a primary pharyngeal lesion.

## References

[1] Abraham SC, Wang H, Wang KK, Wu TT (2008). Paget cells in the esophagus: assessment of their histopathological features and near-universal association with underlying esophageal adenocarcinoma. Am J Surg Pathol.

[2] Nonumara A, Kimura A, Mizukami Y (1993). Paget’s disease of the esophagus. J Clin Gastroenterol.

[3] Matsukuma S, Aida S, Shima S (1995). Paget’s disease of the esophagus. A case report with review of the literature. Am J Surg Pathol.

[4] Haleem A, Kfoury H, Al Juboury M (2003). Paget’s disease of the oesophagus associated with mucous gland carcinoma of the lower oesophagus. Histopathology.

[5] Karakök M, Aydin A, Sari I (2002). Paget’s disease of the esophagus. Dis Esophagus.

[6] Kuan SF, Montag AG, Hart J, Krausz T, Recant W (2001). Differential expression of mucin genes in mammary and extramammary Paget’s disease. Am J Surg Pathol.

[7] Westwood DA, Macemon JB, Coulter GN, Chalmers-Watson TA, Roberts RH (2011). Primary oesophageal malignant melanoma. J Gastrointest Surg.

